# Relation between Microstructures and Macroscopic Mechanical Properties of Earthen-Site Soils

**DOI:** 10.3390/ma15176124

**Published:** 2022-09-03

**Authors:** Yingmin Zhang, Guang Yang, Wenwu Chen, Lizhi Sun

**Affiliations:** 1 CCCC First Harbor Consultants Co., Ltd., Tianjin 300222, China; 2 Department of Civil & Environmental Engineering, University of California, Irvine, CA 92697-2175, USA; 3 China Water Resources Beifang Investigation, Design & Research Co., Ltd., Tianjin 300222, China; 4 Key Laboratory of Mechanics on Disaster & Environment in Western China, School of Civil Engineering & Mechanics, Lanzhou University, Lanzhou 730000, China

**Keywords:** earthen-site soils, consolidated soils, sphericity, porosity, finite element method

## Abstract

While the macroscopic mechanical properties of earthen-site soils have undergone extensive experimental and modeling studies, few research efforts focus on the relationship between the overall mechanical behavior and micro-pore structure. We developed a microstructure-based finite element model to investigate the influence of micro-pore structure on the macroscopic mechanical behavior of earthen-site soils. Scanning electron microscopy images of the untreated and consolidated soils were processed to compare the changes in equivalent diameter, sphericity, and porosity of the soils after consolidation. According to the pore parameter range of the untreated and consolidated soils, the effects of micro-pores on the soil behavior are specifically conducted under both static and dynamic loads. The relationships between pore characteristics and stiffness, strength, and ultrasonic wave velocity are established.

## 1. Introduction

**Figure 1:**
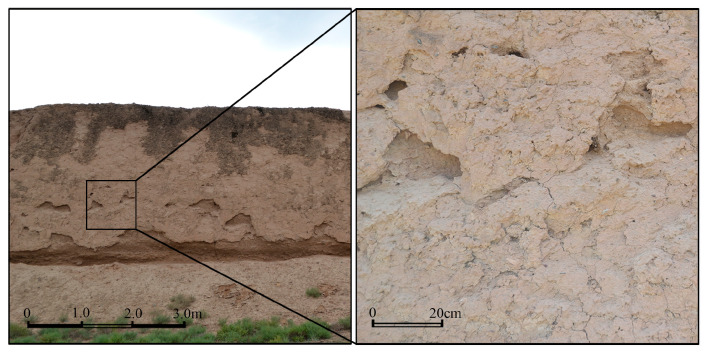
Deterioration in earthen sites of the Ming Dynasty Great Wall in Yongchang County, Gansu Province, China.

Earthen sites are evidenced all over the world [1]. Earthen-site soils as primary construction materials have been widespread since ancient times in many areas of Africa, Asia, and Central and South America. About 30% of the world’s population still lives in earthen buildings as per the United Nations [2]. The outdoor immovable earthen sites are located in complex environmental conditions, exposed to the natural environment for a long time, with their surfaces severely weathered (Figure 1). Consolidation may be imperative to promote and restore the strength of the earthen-site soils.

Research efforts on the protection of earthen sites have aroused wide interest since the 1960s. While remarkable advances have been made in the consolidation and repair of earthen sites, the conservation of earthen sites is still a great challenge, with surface weathering being one of the critical issues to be further solved.

The research on anti-weathering materials is increasingly prosperous. There are many kinds of anti-weathering materials, which can be divided into organic materials, inorganic materials, and organic–inorganic hybrid materials according to their chemical compositions. Organic materials have significant improvement in stone artefacts due to their favorable permeability and adhesiveness [3,4,5]. Acrylic polymeric resins and silicon-based strengthening agents are used for the conservation of monumental buildings and other porous materials [6,7,8,9]. Inorganic materials have strong aging resistance, low cost, and suitable compatibility with ancient ruins [10]. New calcium alkoxides reacted with atmosphere to generate calcium carbonate, which was used to consolidate carbonate rocks [11,12]. Alkaline solutions for the consolidation of earthen structures revealed a positive change in water resistance and mechanical strength [13]. Barajas et al. [14] compared the physicochemical effects of organic and inorganic consolidants on the Tlaltecuhtli monolith. Innovative phosphate-based consolidants were also compared with a commercial ethyl silicate in terms of effectiveness and compatibility [15]. Zhang et al. [16] found that the consolidation effect of combined materials on earthen-site soils is better than that of single materials in accordance with laboratory results.

The change in mechanical properties of soils after treatment is one of the core issues in evaluating improvement effects. The mechanical properties of soils can be divided into two categories, namely static properties and dynamic properties. The static properties of soils include stiffness, strength, constitutive relations, etc. Uniaxial compressive strength is the peak stress of the material resistance to axial compression when the load is slowly applied along the axis at a certain rate without confining pressure, which can directly indicate the strength of the material. The dynamic properties of soils include dynamic strength, dynamic constitutive relations, vibration characteristics, wave characteristics, etc. The nondestructive ultrasonic technique is prevalent in the field of cultural relic protection because of its small interference with the detected object. Previous studies have suggested the relationship between strength and acoustic parameters [17,18]. An exponential relation exists between unconfined compressive strength (UCS) and ultrasonic pulse velocity (UPV) for materials, such as foamed mixture lightweight soil [19] and concrete [20,21,22]. Tan et al. [23] established mathematical models of coral aggregate concrete to explore the effects of sisal and age on the relationship between UCS and UPV. A linear correlation between UCS and UPV was found for foam-cemented paste backfill [24] and carbonate rocks [25]. The UPVs of most of consolidated earthen-site soils decrease to a certain extent compared to the untreated soils, while their UCSs increase [16]. Reasons for the different changes in strength and acoustic properties are not well understood. Attempts thus far have been made to explore the relationship between them from statistics, without considering the microstructure of materials. Few studies were carried out to reveal the quantitative relationships between UCS, UPV, and pore characteristics; thus, it is difficult to predict the engineering properties of materials.

The change in physical and mechanical properties of materials is the result of the interaction of their composition [26,27], micromechanical properties, and microstructure [28,29]. The microstructure of materials can be observed by environmental scanning electron microscopy (ESEM) [30] and mercury intrusion porosimetry (MIP) [31]. The combination of ESEM and digital image correlation (DIC) technology can determine the deformation and failure mechanism of mudstone and consolidated earthen-site soil from the microscopic scale [32], investigate the influence of humidity on the mechanical behavior of mudstone [33], and perform full-field strain measurements for low-strain geomaterials [34]. Zhao et al. [35] analyzed the influence of microstructure on fracture mode by using image processing and analysis techniques. Liu et al. [36] fitted the expression of correlation between pore size and UCS of concrete.

The recent threads of applying computed tomography (CT) and scanning electron microscopy (SEM) have promoted the development of real microstructures, which has also laid the foundation for the study of the relationship between the microstructure and mechanical behavior of consolidated earthen-site soils. While previous studies focus on explaining the macroscopic physical and mechanical properties of materials from the perspective of the microstructure, the macroscopic mechanical analysis based on microscopic images fails to consider the influence of the microstructure parameters, thus failing to clarify the essential causes of deformation and failure. With the rapid development of computer technology, the study of mechanical behavior extends from laboratory and field tests to numerical simulation. Particle agglomerates modeled by particle flow codes were used to investigate the effects of size [37], porosity, and friction coefficient [38] on strength properties of soils. The two-dimensional particle assembly model can study the influence of contact laws [39], particle shape [40], and particle breakage [41,42,43] on mechanical parameters, and simulate various laboratory tests to capture the macroscopic behavior of granular media [44,45,46]. Nguyen et al. [47] undertook simulations of concrete with an X-ray CT image-based model to study the failure mechanism during in situ compression. Zhang et al. [48] visualized the pore distribution of consolidated earthen-site soils and simulated the UCS test by the finite element method, but did not consider the influence of pore characteristic parameters. In addition, stress wave propagation can be simulated by the finite element model [49]. Numerical simulation of ultrasonic pulse effectively detected the early-stage corrosion of steel rebar [50], revealed the physical mechanisms of wave modulus of elasticity as being higher than the static modulus of elasticity through sinusoidal wave and compression testing techniques [51], and correlated the ultrasonic parameters with the elastic modulus, UCS, and density of the materials [19].

Due to complex structure of the earthen-site soils, the quantitative relationships between pore parameters and macroscopic properties are not well understood. Traditional testing methods lack quantitative investigation of the relationship between micro-pore parameters and strength, stiffness, and acoustic characteristics of soils; hence, it is difficult to directly and comprehensively analyze the reasons for the changes in the mechanical properties of the consolidated earthen-site soils. After the introduction of numerical simulation, the above-mentioned problems are expected to be well solved. The numerical model can strictly control the variables and conform to the principle of reducing intervention in the cultural heritage conservation criteria. Micromechanics-based finite element simulation may provide a new route for exploring the causes of macroscopic phenomena.

In the current study, we aimed to develop a physics-based numerical model for quantifying the relationship between micro-pore structure characteristics, strength, and acoustic parameters of earthen-site soils. A microstructure-based finite element model was established to control the change in pore parameters. The model reveals how pore equivalent diameter, pore sphericity, and porosity affect the macroscopic mechanical properties of soils under linear axial load and harmonic pulse load. This study analyzes the reasons for the variations in unconfined compression strength (UCS) and ultrasonic pulse velocity (UPV), as well as deepens the understanding of soil improvement. The porosity has a great influence on the macroscopic mechanical behaviors of soil. The compression wave velocity of the non-porous medium is more than twice that of the medium with 40% porosity. The quantitative investigations between the macroscopic performance and microscopic characterization relationships will be helpful to guide the design and construction of cultural relic conservation projects.

## 2. Materials and Methods

### 2.1. Image Processing and Characterization of Pores

The purpose of quantifying the microstructure of soils is to acquire the sphericity, size, quantity, distribution location, fractal dimension, and other indexes of particles or pores. In order to determine these parameters, it is necessary to observe the morphology of soils qualitatively by using microstructure testing equipment to obtain microstructure images, followed by extracting microstructure parameters through a series of image processing and analysis procedures.

**Figure 2:**
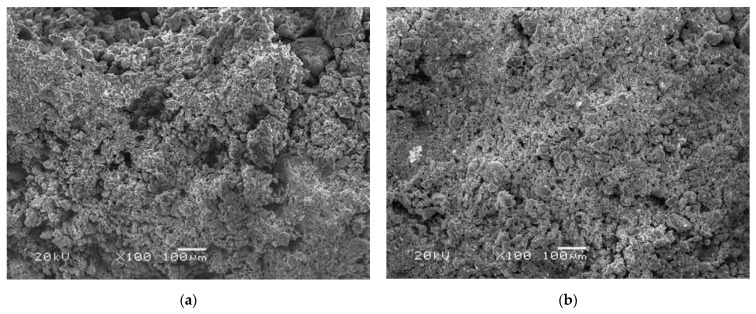
Scanning electron microscopy images: (**a**) untreated sample and (**b**) consolidated sample.

A published study [16] indicated that cubic samples consolidated by Bio Line^®^ Ethyl silicate+Bio Line^®^ Micron lime have obvious advantages over other materials in terms of physical and mechanical properties. Therefore, scanning electronic microscopy (SEM) images with 100× magnification of consolidated and untreated samples were selected for microstructure analysis in this study (Figure 2). The untreated and consolidated earthen-site soils are regarded as a two-phase system consisting of a solid (matrix) and a gas (pore). The quantization process of the images’ microstructure parameters can be divided into four steps. The first step is to format the images to BMP files and import them into image processing software for segmentation. The second step is to preprocess the images before segmentation. The images were filtered to reduce the noise, as it would interfere with the observation of information. Thereafter, the artifacts were removed, and the features of interest were enhanced. The third step is to identify the interface between different substances based on pixel intensity. The pre-processed images were separated into different regions—matrix and pores—by the threshold segmentation method [35,52]. Then, isolated pixels or pixel blocks were removed and the nodes that had little influence on the particle morphology were reduced to improve the computational efficiency. The fourth step is parameter extraction. The porosity of samples was calculated according to the area ratio of each region. Parameters such as pore equivalent diameter and sphericity were obtained by image analysis module.

### 2.2. Selected Area and Threshold Settings

Threshold has a great influence on modeling. Different threshold values were set for images in the range of 70–125 with 5 as threshold interval to determine the optimal threshold value. Porosities of soils were calculated as per their area proportion, and the relationship between porosity and threshold was ascertained.

In addition, the study area should be representative due to the heterogeneity of microstructure of soils. The image should contain enough particles and pores. The pixel size of the SEM image with 100× magnification is 960 × 1280. Eight selection areas, i.e., 120 × 160, 240 × 320, 360 × 480, 480 × 640, 600 × 800, 720 × 960, 840 × 1120, and 960 × 1280, were set to establish the relationship between threshold and porosity so as to determine the optimal selection area.

### 2.3. Simulations of Linear Axial Load Transfer in Soils

Simulations were carried out on Marc Mentat 2020.0.0 developed by MSC software Corporation. The soil is an inhomogeneous medium with complex and diverse particle composition and pore structure. In continuum mechanics, constitutive relations are normally applied to study the responses of uniform media to loads. Therefore, heterogeneous soil can be equivalent to uniform material, and the representative volume element (RVE) should be selected.

The samples and loads in the process of uniaxial compression were symmetric. Only half of the model was selected, and the left boundary was taken as the symmetric boundary. Therefore, the horizontal displacement of the left boundary was constrained to zero. The geometric properties of the elements were structural plane stress. The vertical displacement was loaded on all nodes of the upper boundary at a loading rate of 0.05 mm/s. The nodes of the lower boundary were fixed, and those of the right boundary were free. The static structural analysis in Marc software was conducted to simulate the mechanical behavior of the soils under linear axial load. As the yield and failure behaviors of soils were involved, large strain was employed for calculation.

The isotropic elastoplastic model was applied to analyze mechanical response of microstructure under axial compression, and the Young’s modulus and Poisson’s ratio of the medium had to be given. The Mohr–Coulomb yield criterion was adopted to describe the plastic properties of soils, and the yield stress of the material had to be defined. The Mohr–Coulomb criterion is a combination of Hooke’s law of isotropic linear elasticity and Coulomb’s law of perfect plasticity. In finite element analysis, the response of force and displacement was determined by the path graph. In post-processing, the vertical stress of a cross-section could be calculated as per the force in the vertical direction as,

(1) $$\sigma =\frac{RF_{2}}{A}$$

where *σ* is the axial stress, *RF*_2_ is the reaction force in the vertical direction, and *A* is the area of the cross-section.

The reaction force in the vertical direction can be calculated by multiplying the stress at each node with the area of element. The formula of stress can be rewritten as

(2) $$\bar{\sigma }=\frac{1}{A}\int \sigma \cdot dA=\frac{\sum_{i=1}^{n}\sigma_{i}A_{i}}{\sum_{i=1}^{n}A_{i}}$$

where $\bar{\sigma }$ is the average vertical stress, $\sigma_{i}$ is the vertical stress of the node, $A_{i}$ is the area of the element, and n is the number of nodes at the cross-section.

The determination of parameters can refer to the range of empirical data due to the limitations of current testing technology. Many simulations and specifications have given the range of Poisson’s ratios for soils. The specific gravity and plasticity index of the earthen-site soils are 2.72 and 10.16, respectively, thus being silty clay according to the *Code for Investigation of Geotechnical Engineering* [53]. Clays are generally assumed to be undrained. In an undrained analysis of cohesive soils such as silty clay, the Mohr–Coulomb criterion is reduced to the well-known Tresca yield criterion. For saturated clay, Poisson’s ratio is in the range of 0.4 to 0.5 and the friction angle is close to 0 [54,55]. Poisson’s ratio was 0.44 in this study, and the Young’s modulus and yield stress of the matrix were 161 MPa and 3.32 MPa, respectively [48]. It is also noted that in the consolidation of heritage/earthen-site soils, the unconfined compressive strength is usually used to analyze the change in the strength of the heritage after consolidation, so as to evaluate the consolidation effect of the consolidants in the literature [10,56]. We studied the effect of different microstructural parameters on strength; therefore, the unconfined compressive strength test was used instead of the triaxial test.

### 2.4. Simulations of Ultrasonic Propagation in Soils

The propagation characteristics of ultrasonic waves are closely related to the property and structure of soils. The ultrasonic detector with two transducers can detect the physical and mechanical properties of soils. One sensor served as a pulse transmitter and the other was used for receiving the pulse. The time of flight of the compression wave ($t_{P}$) and shear wave ($t_{S}$) is measured by the input and output signals. According to the distance between the transmitting position and receiving point (*l*) and the time of flight of compression wave ($t_{P}$) and shear wave ($t_{S}$), ultrasonic wave velocities can be calculated by

(3) $$v_{P}=\frac{l}{t_{P}}$$

(4) $$v_{S}=\frac{l}{t_{S}}$$

where $\nu_{P}$ is the propagation velocity of the compression wave, and $\nu_{S}$ is the velocity of the shear wave. Furthermore, the dynamic shear modulus of the material ($G_{d}$) can be assessed according to the calculated velocity of the shear wave:

(5) $$G_{d}=\rho ‧v_{S}^{2}$$

where *ρ* is the density of the material.

Based on the assumption that the medium is homogeneous, isotropic, and elastic, the dynamic elastic parameters of the material can be computed in accordance with the ultrasonic wave velocity:

(6) $$E_{d}=\frac{\rho v_{s}^{2}\left(3v_{P}^{2}-4v_{S}^{2}\right)}{v_{P}^{2}-v_{S}^{2}}$$

(7) $$\nu_{d}=\frac{v_{p}^{2}-2v_{s}^{2}}{2\left(v_{P}^{2}-v_{s}^{2}\right)}$$

where $E_{d}$ is the wave modulus of elasticity, and $\nu_{d}$ is the dynamic Poisson ratio.

Three-node triangle elements were used in the meshing. The dynamic transient structural analysis in Marc software was conducted to simulate the mechanical behavior of the soils under simple harmonic pulse. Only small elastic deformation occurs in soil because of the low energy of ultrasonic waves. The soil can return to its original state after removing the load; thus, it belongs to small strain.

A half-sine pulse with a frequency of 50 kHz was applied at one boundary of the RVE and transmitted signals were received at its opposite boundary. Four positions were selected for transmitting and receiving signals. The ultrasonic wave was excited from the top edge of RVE and received from the bottom edge, marked as TB. The signal was excited from the bottom edge of RVE and received from the top edge, named BT. The half-sine excitation was loaded to the nodes on the left boundary and output from the right boundary, marked as LR. The pulse was loaded to the nodes on the right boundary and output from the left boundary, named RL. Time of flight was estimated by subtracting a quarter period of input signal from the time corresponding to the first peak of transmitted signal, and then determining ultrasonic wave velocity according to Equations (3) and (4). The wave modulus of elasticity of the matrix was 161 MPa, the dynamic Poisson ratio was 0.44, and the mass density was 1.605 g/cm^3^.

### 2.5. The Establishment of Models

Finite element models of isotropic homogeneous medium with pores were developed, in which the pores were randomly distributed and of regular shape, and the size and shape of pores in the same medium were completely consistent. The micromechanics-based model including simple pores could control single variables easily, and explore the effect of structural characteristics on strength, stiffness, and acoustic parameters of soils. The pore equivalent diameter, pore sphericity, and porosity of medium were set to a series of gradient values to observe their influence on mechanical behavior. The static behavior of the medium was achieved by applying linear axial displacement to the model, while the dynamics of the medium were obtained by loading sinusoidal acceleration pulses.

## 3. Results

### 3.1. Selected Area and Threshold Optimization

**Figure 3:**
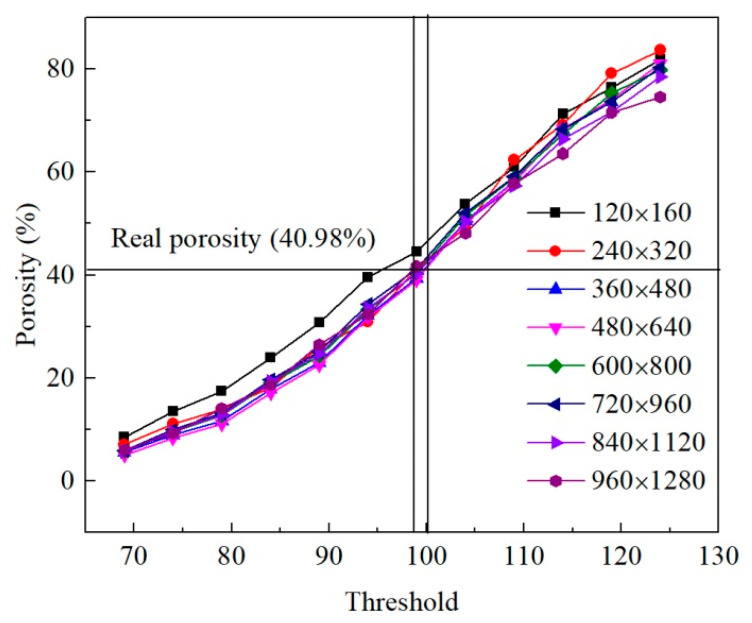
Relationship between porosity and threshold in different selection areas.

Figure 3 illustrates that the porosity of the image rose with the increase in threshold. The porosity of the selected area with 120 × 160 pixels was much higher than that of other areas at a smaller threshold. The 240 × 320 precinct had a slightly larger porosity at a larger threshold. The smaller selected area contains less information and is not enough to represent the microstructure of soil. The selected area with 960 × 1280 pixels had a low porosity at a larger threshold. This is because the SEM image contains annotations such as voltage, magnification, and scale, which are identified as soil during image segmentation. Therefore, this selected area had errors due to the interference of annotation information.

**Figure 4:**
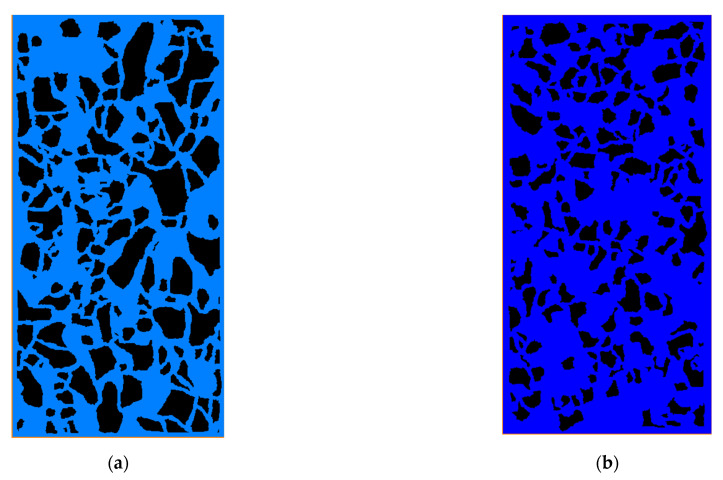
Representative volume element (RVE) of (**a**) untreated sample and (**b**) consolidated soil.

The macroscopic porosity of the untreated sample measured by the geotechnical test was 40.98%, and its corresponding threshold range was 99–100. The thresholds were set to 99 and 100, respectively, and the errors between micro and macro porosity of each selected area were calculated as shown in Table 1. In the range of 99–100, the deviations of micro and macro porosity were all within 5%, except for the selection area of 120 × 160. The porosities of selected areas with 720 × 960, 840 × 1120, and 960 × 1280 were interfered by annotations of SEM images. The selected areas with 360 × 480, 480 × 640, and 600 × 800 were, therefore, more representative. In general, the error of the porosity with a threshold of 100 was less than 99. Therefore, the threshold was set to 100 with deviations of less than 2.5%, which met the requirements of microstructure analysis. Under the condition that porosity requirements are satisfied, the pixel should be selected to be as large as possible so that it contains enough particles to improve the accuracy of analysis. Since the mechanical behavior of the cubic sample under linear axial displacement load is symmetrical, half of the sample can be selected as RVE when building the finite element model. In addition, the labeling information needs to be avoided, and the largest area that can be selected at this time is 640×1280. The obtained RVEs of the untreated and consolidated samples are shown in Figure 4. The width and height of the RVE are 6.4 mm and 12.8 mm.

### 3.2. Microstructural Characterization of Soils

**Figure 5:**
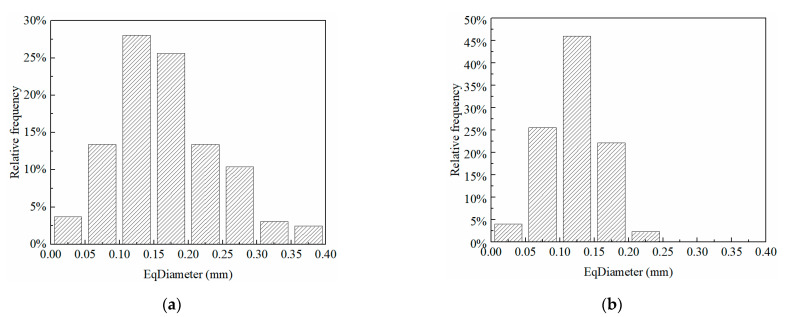
The equivalent diameter distribution of pores: (**a**) untreated sample; (**b**) consolidated sample.

It is noted that while rigorous modeling and simulation can be executed, based on the real pore profile (Figure 4) to determine the macroscopic performance of soils, it is challenging to conduct a quantitative investigation on how such microstructure affects the overall mechanical properties of the soils, since Figure 4 cannot provide direct quantitative parameters. Therefore, in this section, the parameters of porosity, equivalent diameter, and sphericity are introduced to characterize the pore structure, based on which the effect of microstructure on overall properties can be systematically conducted, using the proposed parameters. The pore equivalent diameter, pore sphericity, and porosity of the untreated and consolidated earthen-site samples can be estimated based on the microstructures from SEM images and image-processing procedures. The equivalent diameter of the untreated sample was larger than that of the consolidated sample in terms of mean, minimum, maximum, and median values (Table 2). The equivalent diameter distribution range of pores in the untreated sample was wider than that in the consolidated sample (Figure 5). The concept of skewness (SK) was proposed to describe the symmetry of a frequency–distribution curve, which can be evaluated by the difference between arithmetic mean and mode. SK = 0 signifies the symmetrically distributed data, SK > 0 indicates the right-skewed data, and SK < 0 represents left-skewed distribution. The equivalent diameter of the untreated sample was right-skewed with a large skew degree, while that of the consolidated sample was left-skewed with a small skew degree. Moreover, kurtosis (Ku) was used to characterize the sharpness of the peak of a frequency–distribution curve. Specifically, Ku = 3 reveals the mesokurtic data. A data value greater than 3 presents a leptokurtic distribution, while a data value less than 3 displays a platykurtic distribution. When skewness is 0 and kurtosis is 3, the data are normally distributed. The equivalent diameters of untreated and consolidated samples followed a platykurtic distribution. The average equivalent diameters of untreated and consolidated samples were 0.12 mm and 0.17 mm, respectively. The equivalent diameter distribution of these two samples ranged from 0.01 mm to 0.39 mm.

**Figure 6:**
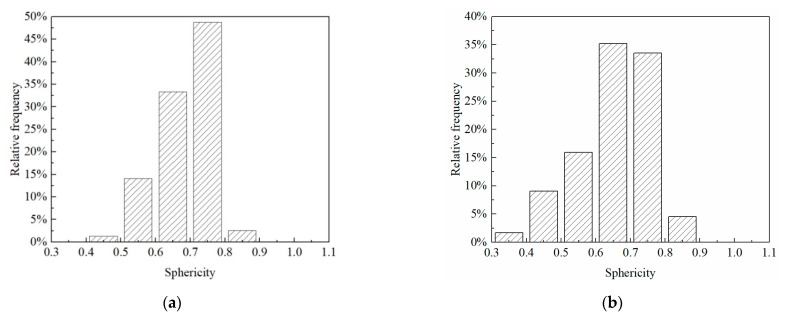
The sphericity distribution of pores: (**a**) untreated sample; (**b**) consolidated sample.

The sphericity of the untreated sample was greater than that of the consolidated sample in terms of mean, minimum, maximum, and median values (Table 3). However, the distribution range of pore sphericity in the untreated sample was smaller than that in the consolidated sample (Figure 6). The pore sphericity of untreated and consolidated samples showed a left-skewed and platykurtic distribution. The average sphericities of pores of untreated and consolidated samples were 0.688 and 0.653, respectively, and their sphericities ranged from 0.349 to 0.851. The porosity of soil can be determined by the ratio of pore area to total area after image segmentation. The porosity of untreated soil was 40.81%, and that of consolidated soil was 26.24%.

### 3.3. Effect of Equivalent Diameter Distribution of Pores on Strength, Stiffness, and Ultrasonic Pulse Velocity of Soils

Pore equivalent diameter is a parameter to characterize the size of pores. It refers to the diameter of an equal-volume sphere in a three-dimensional system. In a two-dimensional system, it can be defined as the diameter of an equal-area circle, as displayed in Equation (8).

(8) $$EqD=\sqrt{\frac{4S}{\pi }}$$

where *EqD* is the equivalent diameter of two-dimensional pores, and *S* is the projected area of pores.

**Figure 7:**
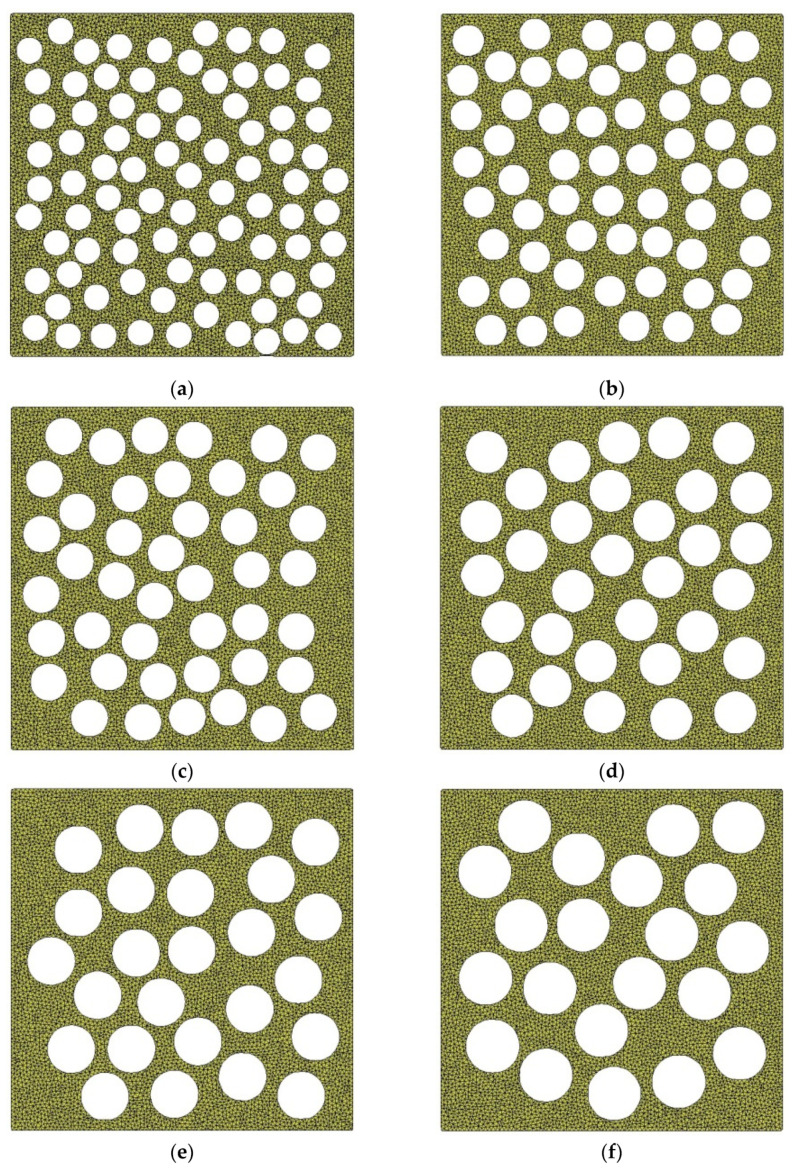
Models with different pore equivalent diameters: (**a**) *EqD* = 0.10 mm; (**b**) *EqD* = 0.12 mm; (**c**) *EqD* = 0.14 mm; (**d**) *EqD* = 0.16 mm; (**e**) *EqD* = 0.18 mm; (**f**) *EqD* = 0.20 mm.

Since the equivalent diameter of pores of earthen-site soils was concentrated in the range of 0.10–0.20 mm, the circular pores with diameters of 0.10 mm, 0.12 mm, 0.14 mm, 0.16 mm, 0.18 mm, and 0.20 mm were employed to compare the effects of different equivalent diameters of pores on macroscopic static and acoustic parameters of the medium (Figure 7). Parameters remained the same in all RVEs except the diameter of pores, and the porosity of the medium was 40.67%. The pores in the same RVE were spherical, equal in size, and randomly distributed.

**Figure 8:**
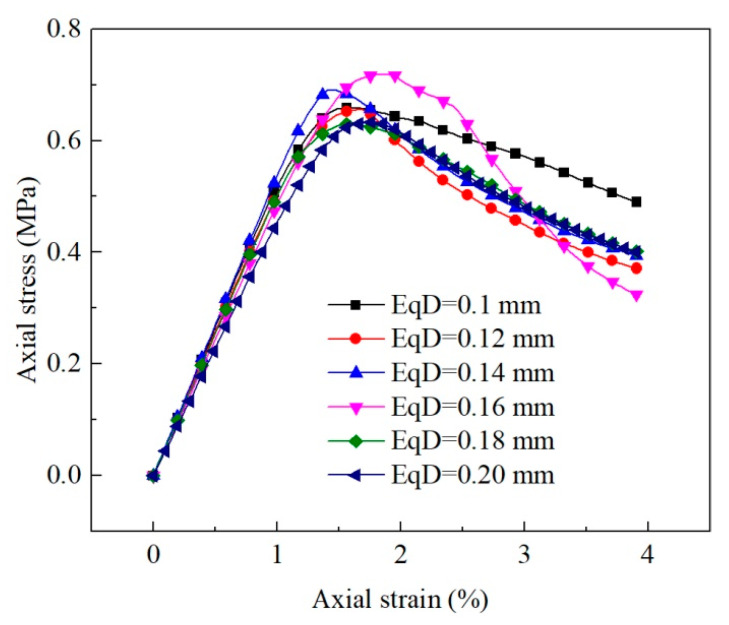
Macroscopic static response of equivalent diameter of pores.

Figure 8 reveals that the equivalent diameter of pores had little influence on the stress–strain curve, and the peak value of the curve generally rose first and then dropped with the increase in equivalent diameter. The average equivalent diameter of pores of the untreated sample was about 0.17 mm, and that of the consolidated sample was 0.12 mm as per the analysis of the microstructure of earthen-site soils. When other conditions remain unchanged, the strength of soil should decrease slightly due to the change in the equivalent diameter after consolidation. However, according to the uniaxial compressive strength obtained in the test, the compressive strength of soil after treatment was greatly improved. Therefore, the effect of equivalent diameter of pores on uniaxial compressive strength is not significant. Other microstructural parameters (porosity and sphericity) may be more effective, as we show in later sections.

**Figure 9:**
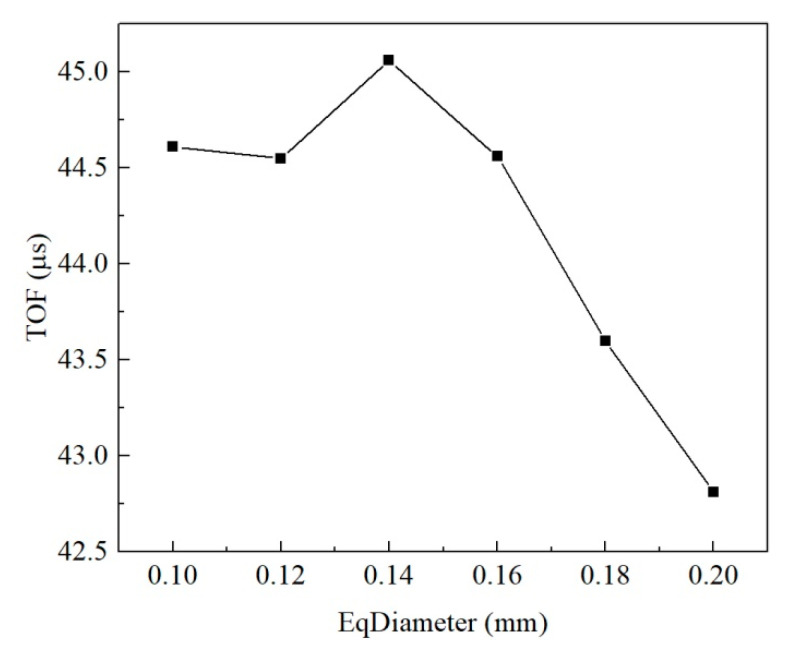
Relation between pore equivalent diameter and time of flight.

The ultrasonic pulse velocity and dynamic elastic coefficient computed from the four loading positions were slightly different. In order to reduce the position error in the results, the wave velocities calculated from the four positions were averaged to study the acoustic characteristics of the medium, and the dynamic elastic characteristics of the medium with different pore equivalent diameters were compared by wave theory. The velocity of the compression wave declined slowly and then increased rapidly as the equivalent diameter of pores increased. Since the average pore equivalent diameters of the untreated and consolidated earthen-site soils were 0.17 mm and 0.12 mm, respectively, the pore equivalent diameter reduced after consolidation, and the transmission time of the half-sine pulse in the medium became longer, resulting in the decrease in the ultrasonic pulse velocity. This may be one of the reasons that the compression wave velocity of soil after consolidation presents a downward trend compared with that before treatment (Figure 9).

In general, ultrasonic wave velocity was less susceptible to pore equivalent diameter. The effect of pore equivalent diameter on shear wave velocity was slightly larger than on compression wave velocity; the variation in dynamic elastic parameters of the medium was thus associated with the change in shear wave velocity. The tendencies of dynamic shear modulus and wave modulus of elasticity happened to coincide with that of shear wave velocity, while the variation trend of the dynamic Poisson ratio with pore equivalent diameter was exactly opposite that of shear wave velocity (Table 4).

### 3.4. Effect of Pore Sphericity on Strength, Stiffness, and Ultrasonic Pulse Velocity of Soils

Pore sphericity is a factor that describes how spherical a pore is. It can be represented by the ratio of the surface area of an equal-volume sphere to the actual surface area of the pores. Two-dimensional pore sphericity can be defined as the ratio of the circumference of an equal-area circle to the actual circumference of the pore, as shown in Equation (9).

(9) $$SI=\frac{2}{L}\sqrt{S\pi }$$

where *SI* is the two-dimensional pore sphericity, and *L* is the pore perimeter.

**Figure 10:**
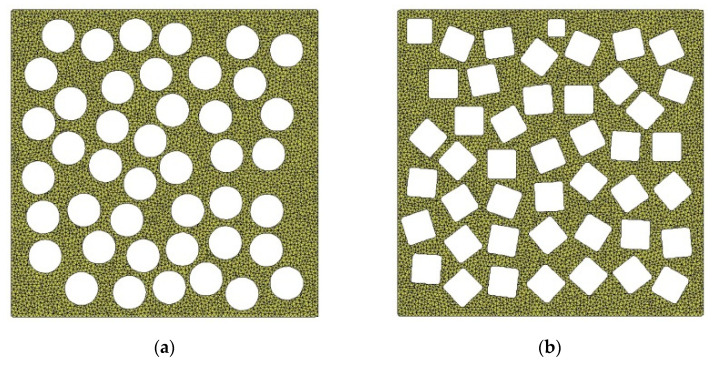

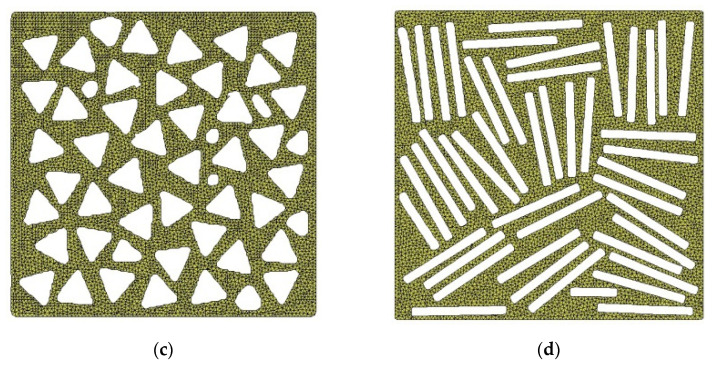
Models with different pore sphericity: (**a**) *SI* = 1; (**b**) *SI* = 0.886; (**c**) *SI* = 0.778; (**d**) *SI* = 0.600.

Pore sphericity is always less than 1. Sphericities of pores of earthen-site soils were mostly in the range of 0.3–0.9; hence, the sphericities of pores were set to 1, 0.886, 0.778, and 0.600 to study the influence of sphericity on the macroscopic static and dynamic behavior of the medium (Figure 10). Parameters of the medium remained the same for all RVEs except for the pore sphericity, the pore equivalent diameter was 0.14 mm, and the porosity of the model was 40.67%. For the same RVE, all pores were identically spherical and randomly distributed.

**Figure 11:**
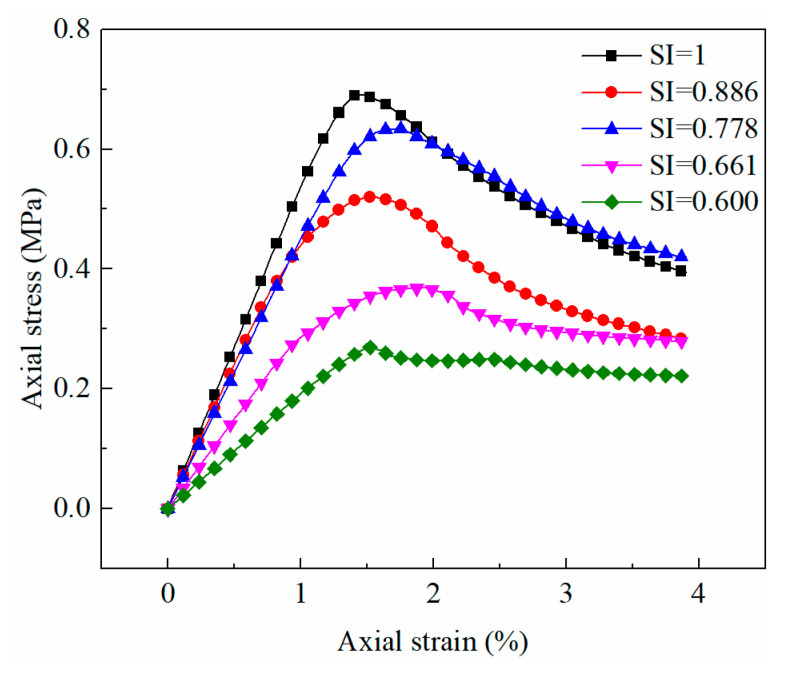
Macroscopic static response of soils as a function of pore sphericity.

Figure 11 demonstrates that the pore sphericity had a significant effect on the stress–strain curves of soils. Secant modulus and peak strength of soil increased with the rise in sphericity. The sphericities of pores of the untreated and consolidated samples were 0.69 and 0.65, respectively. The pore sphericity of the consolidated sample increased compared to that of the untreated sample, which would improve the uniaxial compressive strength of the soil.

The simple harmonic vibrations received by the four loading positions were mildly different. The times of flight (TOFs) of four positions were averaged to characterize the acoustic characteristics of the medium. The larger the sphericity of the pore was, the longer the transmission time of the half-sine wave in the soil was, indicating the decrease in compression wave velocity (Table 5). The sphericities of pores of the untreated and consolidated samples were 0.69 and 0.65, respectively. Under the same condition, the pore sphericity became smaller, and the time of flight of the half-sine pulse in the medium became shorter, thereby resulting in the rising compression wave velocity.

**Figure 12:**
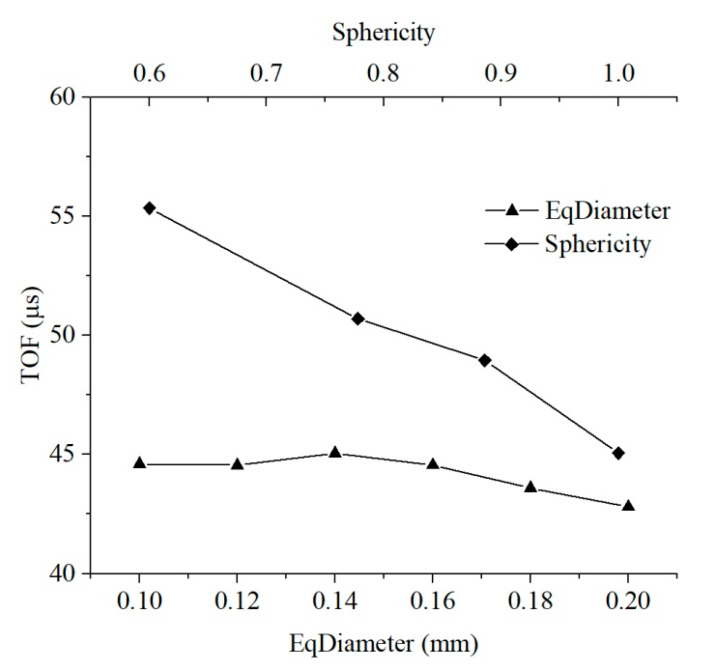
Relationship between sphericity, equivalent diameter, and time of flight (TOF).

The average pore equivalent diameter of soil decreased after consolidation, and the time of flight increased, leading to a decline in the compression wave velocity. However, the drop in the average sphericity of pores resulted in a rise in the compression wave velocity. Therefore, ultrasonic pulse velocity is related to the structural characteristics of pores, and the change in ultrasonic pulse velocity of soils after treatment with different consolidants should be combined with their specific microscopic pore parameters. The relationship between pore parameters and TOF is plotted in Figure 12 to compare the sensitivity of acoustic parameters of media to the equivalent diameter and sphericity of pores. The equivalent diameter had little influence on TOF, while the rise in sphericity contributed to a sharp decline in TOF. The acoustic parameters of the medium were more sensitive to sphericity than to the equivalent diameter of soil within the range of pore parameters studied in this paper.

### 3.5. Effect of Porosity on Strength, Stiffness, and Ultrasonic Pulse Velocity of Soils

**Figure 13:**
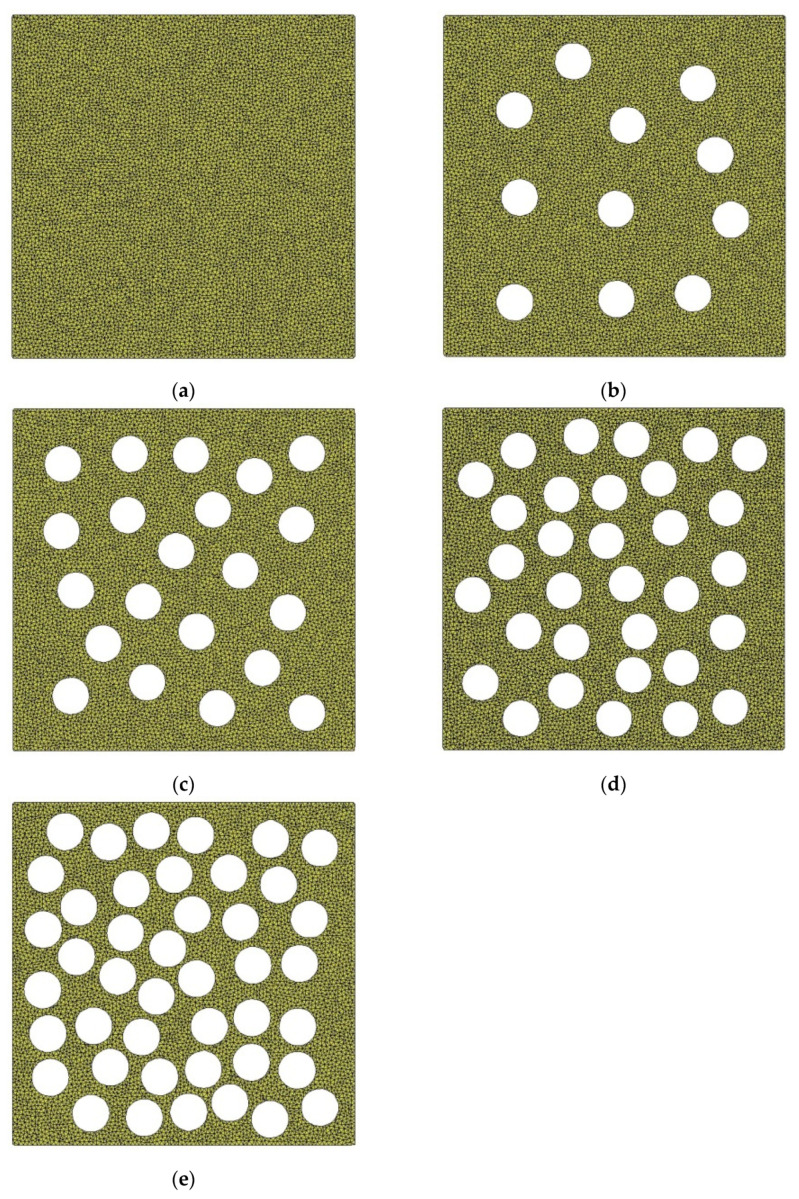
Models with different porosities: (**a**) n = 0; (**b**) n = 10.23%; (**c**) n = 19.27%; (**d**) n = 29.15%; (**e**) n = 40.67%.

Porosity is a measure of the total amount of pores. Area porosity is defined as the ratio of the projected area of pores to the total area of the object. The porosities of the untreated and consolidated soils were in the range of 20–45%. The porosities of the medium were set to 0, 10.23%, 19.27%, 29.15%, and 40.67% (Figure 13). Parameters remained the same for all RVEs except for the porosity, and the equivalent diameter of the circular pore was 0.14 mm. For the same RVE, all pores were identical and randomly distributed.

**Figure 14:**
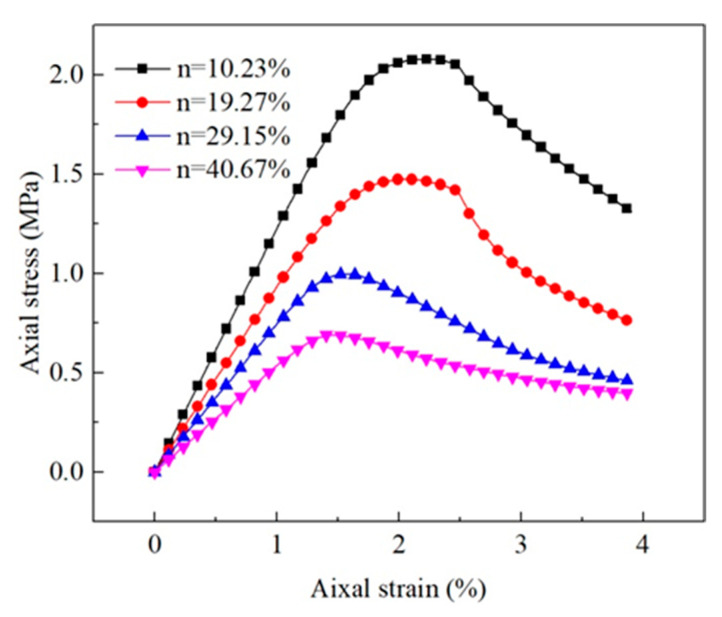
Macroscopic static response of soils as a function of porosity.

**Figure 15:**
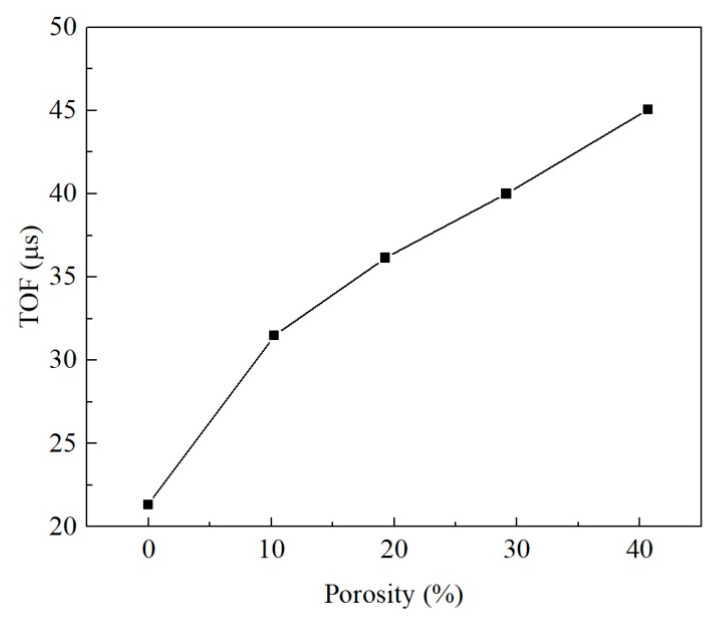
Relationship between porosity and time of flight (TOF).

Porosity had a significant effect on the stress–strain curve of soil (Figure 14). The secant modulus and peak strength of soil decreased greatly with the increase in porosity. The stress of the soil with large porosity reached the yield limit at a small axial strain. The porosities of untreated and consolidated soils were 40.81% and 26.24%, respectively. The decline in porosity after consolidation is one of the important reasons for the increase in the unconfined compressive strength of soil. The time of flight (TOF) of the half-sine pulse in the soil rose drastically with the increase in porosity, and thus, the compression wave velocity reduced (Figure 15). The difference in porosity caused a large difference in ultrasonic pulse velocity, and the compression wave velocity of the non-porous matrix was more than twice that of the untreated earthen-site soil (Table 6). The drop in porosity after consolidation would result in the rise in ultrasonic pulse velocity.

## 4. Discussion

**Figure 16:**
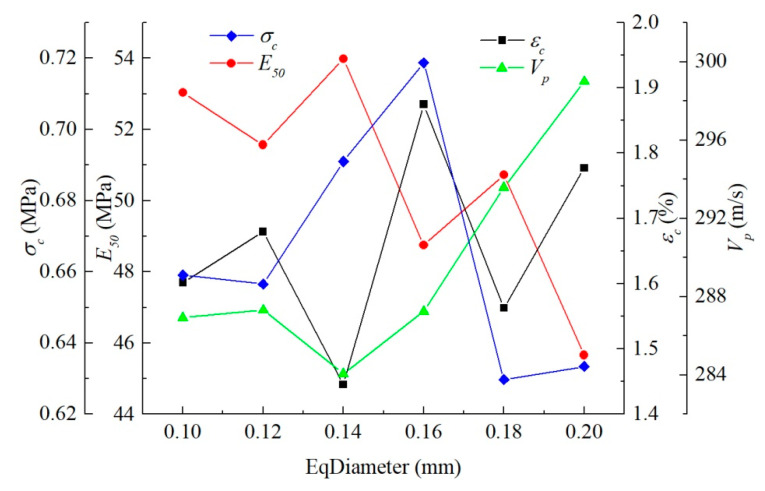
Relationship between pore equivalent diameter and macroscopic static and dynamic parameters.

The pore equivalent diameter has little influence on the strength and stiffness characteristics of the medium, and the macroscopic stress–strain curves of the medium almost coincide (Figure 8). The static parameters of the medium fluctuate in a small range with the increase in pore diameter, indicating that the static behavior of the medium is nearly unaffected by the pore equivalent diameter, and the subtle difference in the macroscopic static behavior is also related to the grid generation of the medium and the distribution of pores (Figure 16). Pore equivalent diameter can influence the macroscopic dynamic behavior of medium. The propagation velocity of the compression wave decreases first and then increases with the rise in pore diameter. The maximum velocity is 1.05 times that of the minimum velocity within the pore equivalent diameter studied in this paper, and the variation is small.

The range of pore equivalent diameter in the actual microstructure of soil is wider; hence, the equivalent diameter greatly changes the propagation velocity of a simple harmonic wave. The average pore equivalent diameter for the untreated soil is 0.17 mm, while that for consolidated soil is slightly reduced to 0.12 mm. The static behavior of the soil does not change visibly after consolidation, and the propagation velocity of the half-sine pulse in the soil decreases in terms of the pore equivalent diameter. Therefore, ultrasonic pulse velocity may decrease when the strength of the soil remains unchanged, which may be one of the reasons why the compression wave velocity of the consolidated soil presents a downward trend in the ultrasound-based test.

**Figure 17:**
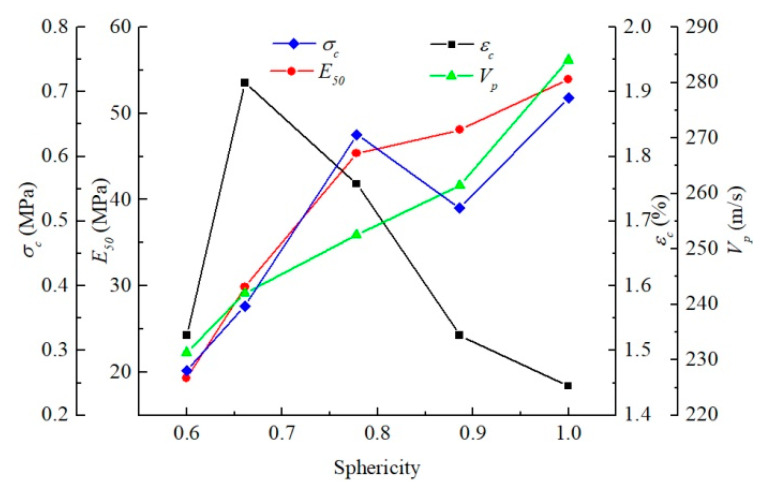
Relationship between pore sphericity and macroscopic static and dynamic parameters.

The pore sphericity has an obvious effect on the static behavior of the medium (Figure 11). The stress–strain curve of the medium changes distinctly with pore sphericity, and the pore sphericity has a considerable influence on the strength and deformation characteristics of the medium. In order to compare the influence of pore sphericity on the macroscopic static and dynamic parameters of the medium more intuitively, the macroscopic mechanical parameters of the medium were extracted to explore their relationship with the pore sphericity of the medium (Figure 17). The unconfined compression strength, secant modulus, and compression wave velocity are positively correlated with the pore sphericity, while the strain at the peak stress is negatively correlated with the pore sphericity. The strength and stiffness of the medium with circular pores are the largest in a two-dimensional structure, and the propagation velocity of simple harmonics is also the fastest. The decline in pore sphericity tends to reduce the strength, stiffness, and propagation velocity of simple harmonics. In the pore sphericity range of 0.600–1, the top strength of the medium is 2.57 times that of the lowest value, the maximum secant modulus of the medium is 2.79 times that of the minimum value, and the fastest propagation velocity of the compression wave in the medium is 1.23 times that of the slowest value. The average pore sphericity of soil after consolidation is 0.6525, and that of the untreated soil is 0.6880. Therefore, the average pore sphericity of soil drops after consolidation, leading to a decrease in the ultrasonic pulse velocity.

For the consolidated soil, the average pore equivalent diameter becomes smaller, the propagation velocity of simple harmonic wave decreases slightly, and the strength and deformation characteristics of soil hardly change. However, its average sphericity decreases, the ultrasonic pulse velocity increases greatly, and the stiffness and strength of soil also increase obviously. Pore sphericity has a more significant effect on the macroscopic static and dynamic parameters of soil than pore equivalent diameter (Figure 16 and Figure 17). Therefore, the macroscopic mechanical properties of the medium are more sensitive to pore sphericity than to pore equivalent diameter within the range of pore parameters studied in this paper.

**Figure 18:**
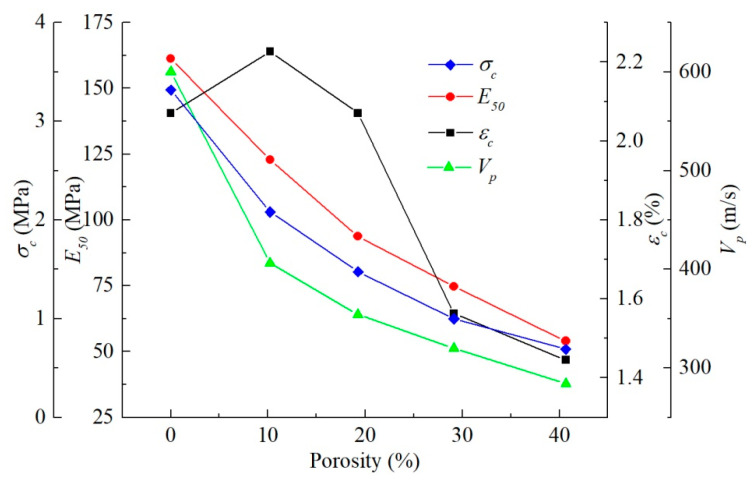
Relationship between porosity and macroscopic static and dynamic parameters.

The macroscopic static behavior of the medium is affected enormously by porosity (Figure 14). The peak stress and secant modulus decrease by roughly the same amplitude with the increase in porosity. As the porosity of the medium rises, the ultrasonic pulse velocity decreases, and the velocity of the medium with 10% porosity decreases significantly compared with that of the non-porous medium. In the range of 10–40% porosity, the variation in the propagation velocity of elastic wave is roughly consistent with the strength and secant modulus (Figure 18). The strain at the peak stress also tends to decrease with the increase in porosity. In the porosity range studied in this paper, the top strength of the medium is 4.80 times that of the lowest value, the maximum secant modulus of the medium is 2.99 times that of the minimum value, the maximum strain at peak stress is 1.43 times that of the lowest value, and the fastest ultrasonic pulse velocity in the medium is 2.11 times that of the lowest value. This demonstrates that the static behavior of the medium is more sensitive to porosity than the acoustic characteristics; hence, the strength and ultrasonic pulse velocity of the soil can be improved by the decrease in porosity after consolidation, and the strength increases more than the velocity.

In terms of the equivalent diameter, sphericity, and porosity, porosity has the greatest influence on the macroscopic mechanical behavior of the medium, followed by sphericity, and the mechanical behavior of the medium is the least sensitive to the pore equivalent diameter. Therefore, the change in porosity and pore sphericity of soil should be paid more attention when studying the macroscopic mechanical properties of soil.

## 5. Conclusions

We developed a microstructure-based model to explore the effect of pore characteristics on strength, deformation, and ultrasonic pulse velocity of earthen-site soils. The main conclusions are drawn based on the presented results and discussion:

1. In the microstructure-based modeling, the selection area of microscopic images should be increased as much as possible. The average pore equivalent diameter, average pore sphericity, and porosity of the untreated sample are larger than those of the consolidated sample.
2. The equivalent diameter of pores has little obvious effect on the static behavior under linear axial load, but is significant on the propagation velocity of the half-sine pulse. With the increase in pore equivalent diameter, the ultrasonic pulse velocity decreases slightly at first, and then rises greatly. The pore equivalent diameter of soil becomes smaller after consolidation, which may be one of the reasons why the compression wave velocity of consolidated soil decreases in the ultrasound-based test compared with that of untreated soil.
3. The sphericity of pores has a significant effect on the static behavior under linear axial load and the acoustic characteristics under harmonic load. The influence of pore sphericity on strength and stiffness characteristics of the medium is greater than on acoustic characteristics. The strength and stiffness characteristics of the medium and the propagation velocity of sinusoidal wave in the medium are positively correlated with the pore sphericity.
4. The porosity has a great influence on the macroscopic mechanical behaviors of soil. The compression wave velocity of the non-porous medium is more than twice that of the medium with 40% porosity. The static characteristics and acoustic parameters of the medium are negatively correlated with porosity.
5. Among the equivalent diameter, sphericity, and porosity, porosity has the greatest influence on the macroscopic mechanical behavior of the earthen-site soils, followed by sphericity. The static behavior and acoustic characteristics of the soils are the least sensitive to the equivalent diameter of pores.

## Figures and Tables

**Table 1:** Comparison of different thresholds and selected areas.

Selected Area	Threshold 99		Threshold 100	
	Porosity (%)	Deviation (%)	Porosity (%)	Deviation (%)
120 × 160	44.50	8.59	46.44	13.32
240 × 320	41.15	0.41	42.92	4.73
360 × 480	39.39	−3.88	41.79	1.98
480 × 640	39.07	-4.66	41.03	0.12
600 × 800	39.42	−3.81	41.90	2.24
720 × 960	40.16	−2.00	42.44	3.56
840 × 1120	40.17	−1.98	42.29	3.20
960 × 1280	41.67	1.68	42.98	4.88

**Table 2:** Comparison of equivalent diameter distribution of pores.

Sample	Mean (mm)	Min (mm)	Max (mm)	Median (mm)	Variance	SK	Ku
Untreated	0.1685	0.0156	0.3863	0.1558	0.0058	0.4818	0.1089
Consolidated	0.1209	0.0125	0.2348	0.1217	0.0018	0.1666	0.1759

**Table 3:** Comparison of sphericity distribution of pores.

Sample	Mean	Min	Max	Median	Variance	SK	Ku
Untreated	0.6880	0.4738	0.8511	0.7024	0.0061	0.7027	0.1261
Consolidated	0.6525	0.3486	0.8375	0.6690	0.0105	0.6805	0.0804

**Table 4:** Macroscopic dynamic response of different equivalent diameters of pores.

Equivalent Diameter (mm)	$v_{p}(m/s)$	$v_{S}(m/s)$	$G_{d}(\mathrm{MPa})$	$\nu_{d}$	$E_{d}(\mathrm{MPa})$
0.10	286.980	165.121	43.778	0.251	109.436
0.12	287.343	161.260	41.931	0.266	105.727
0.14	284.150	171.357	47.145	0.214	114.438
0.16	287.990	154.697	38.486	0.293	99.641
0.18	293.629	153.474	37.815	0.312	99.163
0.20	299.145	171.697	47.315	0.254	118.662

**Table 5:** Influence of pore sphericity on compression wave velocity.

Sphericity	TOF of TB (μs)	TOF of BT (μs)	TOF of LR (μs)	TOF of RL (μs)	Average TOF (μs)	$v_{p}(m/s)$
1	46.10	44.50	440	45.65	45.06	284.050
0.886	49.60	48.20	48.40	49.65	48.96	261.425
0.778	50.65	51.10	51.45	49.55	50.69	252.528
0.600	54.95	56.65	52.25	57.50	55.34	231.308

**Table 6:** Macro dynamic response of porosity.

Porosity (%)	TOF of TB (μs)	TOF of BT (μs)	TOF of LR (μs)	TOF of RL (μs)	Average TOF (μs)	$v_{p}(m/s)$
0	21.35	21.35	21.30	21.30	21.33	600.235
10.23	32.75	31.75	30.70	30.80	31.50	406.349
19.27	36.40	36.45	35.85	35.90	36.15	354.080
29.15	40.30	40.75	39.75	39.20	40.00	320.000
40.67	46.10	44.50	44.00	45.65	45.06	284.050

## Data Availability

The data used to support the findings of this study are available from the corresponding authors upon reasonable request.
